# Prevalence and Baseline Clinical Characteristics of Eosinophilic Chronic Obstructive Pulmonary Disease: A Meta-Analysis and Systematic Review

**DOI:** 10.3389/fmed.2019.00282

**Published:** 2019-12-10

**Authors:** Hong-Xia Wu, Kai-Quan Zhuo, De-Yun Cheng

**Affiliations:** ^1^Department of Respiratory and Critical Care Medicine, West China Hospital, Sichuan University, Chengdu, China; ^2^Department of Neurosurgery, Suining Municipal Hospital of TCM, Suining, China

**Keywords:** eosinophil, chronic obstructive pulmonary disease, biomarkers, inflammation, smoking, airflow

## Abstract

**Background:** Chronic obstructive pulmonary disease (COPD) is a heterogeneous disease with different clinical and pathophysiological characteristics. Cumulative evidence shows that eosinophil levels may be connected to the therapeutic effects and phenotype of COPD. However, the prevalence of eosinophilic inflammation in COPD and the baseline characteristics of eosinophilic COPD remain unknown. Our study investigated the prevalence of COPD with eosinophil levels of >2% and the characteristics of eosinophilic COPD.

**Methods:** We searched the Cochrane Central Library, Medline, Embase, and the Web of Science for trials of eosinophil and COPD published from database inception to May 1, 2019.

**Results:** In total, 40,112 COPD patients that were involved in 19 trials were included in the final analysis. The prevalence of eosinophilic COPD ranged from 18.84 to 66.88%, with an average prevalence of 54.95% across all studies. We found that men, ex-smokers, individuals with a history of ischemic heart disease, and individuals with a higher body mass index (BMI) were at higher risk of eosinophilic COPD (OR 1.36, 95% CI 1.26–1.46, *P* < 0.00001; OR 1.23, 1.12–1.34, *P* < 0.0001; OR 1.31, 1.14–1.50, *P* = 0.001; MD 0.70, 0.27–1.12, *P* = 0.001). There was, however, a lower proportion of GOLD stage I patients among those with eosinophilic COPD (OR 0.84, 0.73–0.96, *P* = 0.01). No significant differences were found in terms of age, current smoker status, pack-years smoked, percent of predicted forced expiratory volume in 1 s, hypertension, diabetes, or other GOLD stages between the two groups (*P* > 0.05).

**Conclusions:** Our analysis suggests that eosinophilic inflammation is prevalent in COPD. Eosinophilic COPD was more likely to occur in men, ex-smokers, those with a higher BMI, and those with a high risk of some comorbidity; however, a lower proportion of patients with eosinophilic COPD experienced mild airflow limitations.

## Introduction

Chronic obstructive pulmonary disease (COPD) is a heterogeneous disease with a variety of features and characteristics. Identification of COPD phenotypes may allow targeted therapeutic strategies. Eosinophilic inflammation is generally believed to be characteristic of asthma, whereas neutrophilic inflammation is considered to be a typical sign of COPD. However, recent reports have shown that eosinophilic inflammation occurs in COPD, in both the exacerbation and stable phases (1, 2). Growing evidence suggests that eosinophil levels may be related to the therapeutic effect and phenotypes of COPD, even after asthma patients are carefully excluded (3–6).

A sputum eosinophil level of >3% is a recognized sign of airway eosinophilic inflammation (4, 6). It was reported that blood eosinophil levels of >2% are indicative of a higher sensitivity in identifying airway eosinophil levels of >3% during COPD exacerbation (1). An alternative cut-off level (≥200 cells per μL or 300 cells per μL) has been used in some studies in addition to the 2% cut-off (7–10). Research has shown that blood eosinophil is a clinically reliable predictor of the inflammatory phenotype. We conclude from these studies that the blood eosinophil level is of reasonable importance in patients with COPD and, as such, is a promising biomarker to guide disease management.

A number of studies have investigated the prevalence and baseline clinical characteristics of patients with eosinophilic COPD. The prevalence of eosinophilic COPD, however, has differed wildly between studies. In one study, 2,083 patients (66%) had eosinophil levels of ≥2% in a *post-hoc* analysis that included 3,177 patients (11). In a retrospective multicenter study enrolling 605 hospitalized patients, 177 patients (29%) had blood eosinophil levels of >2% (12). In a retrospective analysis of a randomized clinical trial, 18.8% of patients had eosinophil levels of >2% (7).

Nonetheless, the baseline clinical characteristics of eosinophilic COPD remain unclear. An analysis of the ECLIPSE cohort study showed that COPD patients with eosinophil levels that were persistently >2% were older, were more likely to be male, were less likely to be a current smoker, had a lower fat-free mass index, and had a higher percent of predicted forced expiratory volume in 1 s (ppFEV_1_) compared with the other COPD groups (2). An observational cohort study suggested that significantly higher numbers of male and young patients were found in the eosinophilic COPD group (13). In a national survey, being male and older in age and having congestive heart failure were significantly associated with eosinophil levels of >2% in COPD (14). In an analysis of the SPIROMICS study, significant differences were found in terms of age, sex, genus, body mass index (BMI), smoking history (pack-years), and current smoker status, but there was no evidence of a difference in the Global Initiative for Chronic Obstructive Lung Disease (GOLD) stage between patients with lower eosinophil (<200 cells per μL) and higher eosinophil (≥200 cells per μL) levels. A significantly lower ppFEV_1_ and FEV_1_: FVC percentage were found in the higher eosinophilic group (8). Couillard et al. (9), however, reported that there was no significant difference between the two phenotypes of COPD in sex, age, smoking status, home oxygen use, comorbidity, lung function, GOLD stage, or hospitalization for COPD in the previous year.

The aim of this study was to evaluate published studies that investigated the prevalence and baseline characteristics of eosinophilic COPD and apply standard meta-analysis methods to gain a more precise result.

## Methods

### Search Strategies

We searched the Cochrane Central Register of Controlled Trials, Medline, the Web of Science, and Embase for studies with the keywords “Eosinophil” and “Chronic obstructive pulmonary disease,” not limited to any language, publication type, or time. We searched for reports published up to May 1, 2019. In order to minimize bias and errors, we also retrieved the reference articles of all included studies. Keywords in related conference articles were also used to retrieve studies. This study was registered with PROSPERO. The findings are reported in compliance with the PRISMA guidelines.

### Inclusion and Exclusion Criteria

Studies matching the following criteria were considered suitable for inclusion: (1) randomized controlled trials (RCTs), as well as observational, cohort, case control, and retrospective studies; (2) trials conducted in patients with COPD aged >40 years; and (3) trials reporting data on the prevalence or baseline clinical characteristics of COPD according to an eosinophil cut-off level of 2% in the blood. Patients admitted due to other medical problems; those with a history of asthma, interstitial pulmonary disease, active pulmonary tuberculosis, or lung cancer; those with other diseases that could influence eosinophil count (eosinophilic pneumonia, allergic diseases, parasitic infections); and individuals with severe dysfunction of other organs or systems or malignant tumors were excluded. Conference articles and trials conducted in pregnant subjects were omitted.

### Outcomes

The prevalence and baseline clinical characteristics of COPD according to eosinophil levels were the primary and the secondary outcomes, respectively. The baseline clinical characteristics of COPD included demographic characteristics (sex, age, and BMI), smoking status (current-smoker, ex-smoker, and pack-years smoked), lung function (ppFEV_1_), comorbidity (ischemic heart disease, hypertension, and diabetes), and GOLD stage.

### Study Selection

Two phases were performed by two separate researchers to verify the studies that met the eligibility criteria. Duplicated studies were first discarded by checking titles and abstracts. Suitable studies were then identified by assessing the full text. Trials reporting data on the prevalence or baseline clinical characteristics of COPD and using an eosinophil cut-off level of 2% in the blood were included.

### Data Extraction

Two researchers extracted suitable information from the included studies following the criteria suggested by Cochrane (15). Corresponding authors were emailed for any missing data.

### Quality Assessment

The Newcastle–Ottawa Scale (NOS) was utilized to evaluate the quality of non-randomized studies (16). Two investigators conducted the quality assessment. A third investigator was consulted to resolve any discrepancies.

### Statistical Analysis

The statistical analysis was performed using the Cochrane systematic review software, Review Manager (RevMan; Version 5.3, The Nordic Cochrane Centre, The Cochrane Collaboration, Copenhagen, 2014). The Mantel-Haenszel test was used to adjudicate statistical significance at a z-value and *P*-value < 0.05, as well as evaluate the hypothesis. The outcomes are shown in forest plots. The outcomes of continuous and dichotomous variables are expressed as mean differences (MD) and odds ratios (OR), respectively. The χ^2^ test with *P* < 0.1 and *I*^2^ > 50% was used to determine significance in the test for heterogeneity. The sensitivity analysis was performed to substitute ranges of values or alternative decisions. A random-effects model was used in case of statistical heterogeneity; otherwise, a fixed-effects model was applied. Any disagreement was resolved by a third investigator reaching a mutual consensus.

## Results

### Study Description

**Figure 1:**
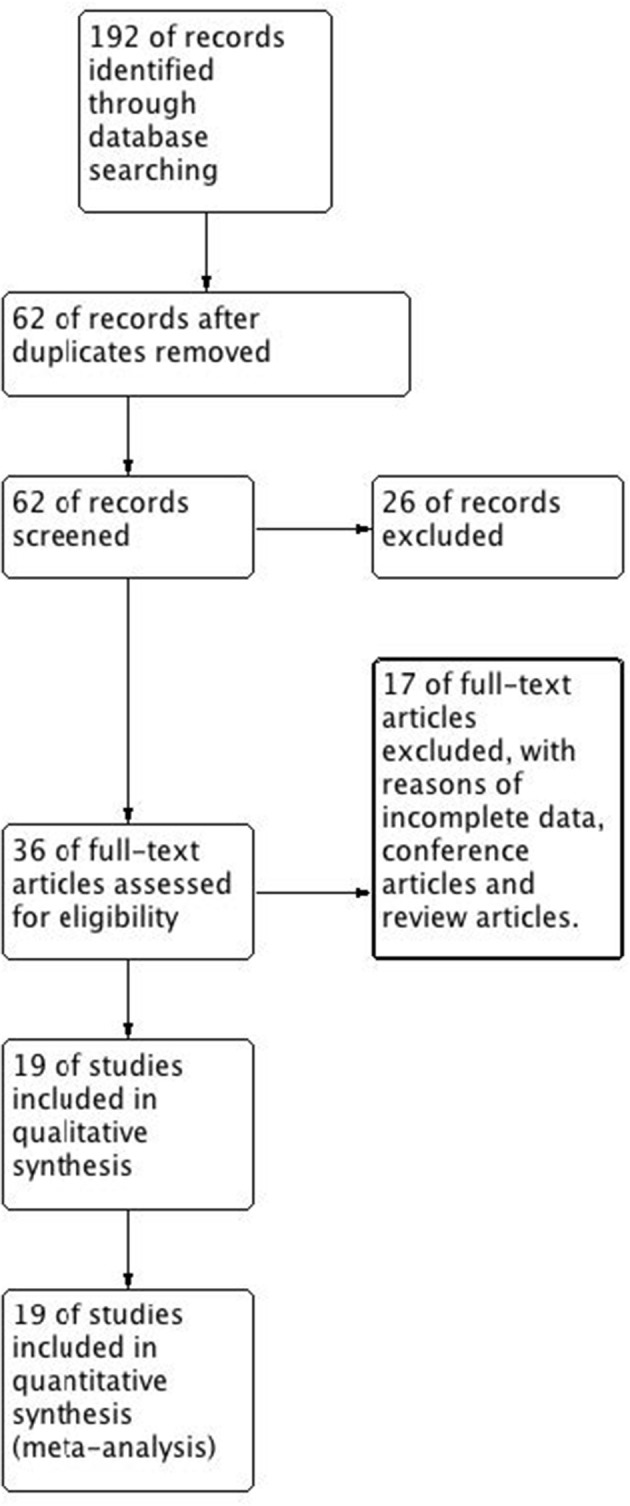
Flow diagram. CENTRAL, Cochrane Central Register of Controlled Trials; RCT, randomized controlled trial.

We searched 192 studies, of which nineteen studies (7–14, 17–27) with 40,112 participants were included in the final analysis (Figure 1). According to the cut-off level of 2% eosinophil in the blood, 22,043 and 18,069 patients were classified as having eosinophilic and non-eosinophilic COPD, respectively. The prevalence of eosinophilic COPD ranged from 9.58 to 66.88%, with a mean of 54.95% among all subjects. The male/female ratios were 15,084:6,959 and 11,363:6,706 in the eosinophilic and non-eosinophilic COPD groups, respectively. The mean age of participants was 62–72 years in the eosinophilic COPD group and 60–73.06 years in the non-eosinophilic COPD group. Regarding the outcomes evaluated, 19 studies (7–14, 17–27) reported data regarding sex, 17 (7–13, 19–27) reported age, 8 (8, 12, 19, 21–23, 26, 27) reported BMI, 15 (7–11, 14, 18, 19, 21–23, 25–27) reported smoking status, 13 (7–11, 17–20, 22, 23, 25–27) reported lung function data, 10 (9, 10, 12–14, 21, 23, 24, 26, 27) reported comorbidities, and 9 reported GOLD stage (9, 10, 12–14, 21, 23, 24, 26, 27). Details of participants' characteristics and outcomes are shown in Tables 1–3. No study was omitted for low quality. The risk of bias assessment is detailed in Table 4.

**Table 1:** Details of each enrolled study.

**References**	**Study type**	**Data source**	**Severity of disease**	**Participants**	**M/F**	**Eosinophils cutoff**	**Eos COPD prevalence %**
Bafadhel et al. (10)	Cohort study	Two-center RCTs	Hospitalized with exacerbation of COPD	243	117/126	200 cells/μL and/or 2%	25.51
Barnes et al. (17)	Cohort study	The ISOLDE study (RCT)	Stable COPD	738	551/187	2%	46.14
Barnes et al. (18)	*Post hoc* analysis	The GlaxoSmithKline Research (RCT)	Stable COPD	6,459	4697/1762	2%	59.44
Çoban Agca et al. (13)	Cohort study	A cohort study	Hospitalized with exacerbation of COPD	1,490	969/521	2%	42.48
Couillard et al. (9)	Cohort study	A multicenter observational clinical trial	Hospitalized with exacerbation of COPD	167	86/81	200 cells/μL and/or 2%	32.93
DiSantostefano et al. (14)	Cohort study	The NHANES (2007–2010)	Stable COPD	948	610/338	2%	66.88
Duman et al. (24)	cohort study	A cohort study	Hospitalized with exacerbation of COPD	1,704	1,116/588	2%	20.6
Hastie et al. (8)	Cohort study	The SPIROMICS cohort study	Stable COPD	2,499	1,361/1,138	200 cells/μL	49.5
Iqbal et al. (19)	*Post hoc* analysis	Four multicenter RCTs	Stable COPD	4,647	3,163/1,484	2%	52.44
Kang et al. (12)	Cohort study	A multicenter retrospective study	Hospitalized with exacerbation of COPD	557	413/144	2%	31.78
Pascoe et al. (11)	*Post hoc* analysis	Two parallel RCTs	Stable COPD	3,177	1,823/1,354	2%	65.56
Pavord et al. (25)	Review	INSPIRE, TRISTAN, and SCO30002	Stable COPD	3,045	2,367/678	2%	65.56
Prins et al. (7)	Cohort study	Two RCTs	Hospitalized with exacerbation of COPD	207	101/106	2% and 300 cell/μl	18.84
Roche et al. (20)	Cohort study	The FLAME study (RCT)	Stable COPD	3,349	2,545/804	2%	61.15
Saltürk et al. (21)	Cohort study	A cohort study	Hospitalized with exacerbation of COPD	647	523/124	2%	9.58
Serafino Agrusa et al. (26)	Cohort study	A case control study	Hospitalized with exacerbation of COPD	132	91/41	2%	15.15
Vedel Krogh et al. (27)	Cohort study	The Copenhagen General Population Study	Stable COPD	7,225	3,719/3,406	2%	63.76
Watz et al. (22)	*Post hoc* analysis	The WISDOM trial (RCT)	Stable COPD	2420	1,989/431	2%	54
Zysman et al. (23)	Cohort study	The Initiatives BPCO French cohort study	Hospitalized with exacerbation of COPD	458	330/128	2%	48.69

*RCT, random-controlled trail; COPD, chronic obstructive pulmonary disease; M/F, male/female; Eos, eosinophilic*.

**Table 2:** Baseline characteristics of patients in each enrolled trial.

**References**	**Subtype**	**Male (*n*, %)**	**Age, years (mean, SD)**	**BMI (mean, SD)**	**Current smoker (*n*, %)**	**Ex-smoker (*n*, %)**	**Pack-years smoked**
Bafadhel et al. (10)	Eos	35 (56.45)	72 (10.25)	NM	14 (22.58)	48 (77.42)	49 (47.5)
	Non-eos	81 (44.75)	71 (12)	NM	42 (23.20)	139 (76.80)	48 (50)
Barnes et al. (17)	Eos	190 (81.55)	63.3 (7.54)	NM	102 (43.78)	NM	44.77 (29.35)
	Non-eos	361 (71.49)	63.94 (6.88)	NM	151 (29.90)	NM	43.39 (32.66)
Barnes et al. (18)	Eos	2,899 (75.51)	NM	NM	NM	NM	NM
	Non-eos	1,797 (68.59)	NM	NM	NM	NM	NM
Çoban Agca et al. (13)	Eos	439 (69.35)	66 (11)	NM	NM	NM	NM
	Non-eos	530 (61.70)	69 (11)	NM	NM	NM	NM
Couillard et al. (9)	Eos	28 (50.91)	69.3 (11.0)	NM	26 (47.27)	29 (52.73)	NM
	Non-eos	58 (51.79)	72.3 (9.8)	NM	63 (56.25)	49 (43.75)	NM
DiSantostefano et al. (14)	Eos	425 (63.03)	NM	NM	201 (31.7)	261 (41.17)	NM
	Non-eos	185 (58.92)	NM	NM	122 (38.85)	112 (35.67)	NM
Duman et al. (24)	Eos	235 (66.9)	70 (4.75)	NM	NM	NM	NM
	Non-eos	881 (65.1)	71 (3.75)	NM	NM	NM	NM
Hastie et al. (8)	Eos	730 (59.01)	65 (3)	28.2 (2.9)	451 (36.46)	NM	45 (6.5)
	Non-eos	631 (50)	65 (3.5)	26.8 (1.8)	522 (41.36)	NM	41 (6)
Iqbal et al. (19)	Eos	1,739 (71.36)	63.5 (8.48)	26.8 (5.69)	1,160 (47.6)	NM	NM
	Non-eos	1,424 (64.43)	63.1 (8.93)	26.7 (5.85)	1,132 (51.22)	NM	NM
Kang et al. (12)	Eos	151 (85.31)	69.89 (11.25)	22.61 (3.49)	53 (29.94)	92 (51.98)	41.39 (26.59)
	Non-eos	262 (68.95)	73.06 (9.34)	21.76 (3.86)	100 (26.32)	168 (44.21)	36.25 (28.76)
Pascoe et al. (11)	Eos	1,232 (59.15)	63.7 (9.25)	NM	NM	NM	NM
	Non-eos	591 (54.02)	63.59 (9.26)	NM	NM	NM	NM
Pavord et al. (25)	Eos	604 (84.1)	64.35 (8.25)	NM	255 (35.47)	NM	36.55 (56.89)
	Non-eos	440 (80)	64.47 (8.41)	NM	228 (41.45)	NM	36.43 (49.44)
	Eos	781 (74.45)	63.41 (8.59)	NM	760 (72.45)	NM	40 (31.46)
	Non-eos	236 (66.69)	62.83 (8.54)	NM	211 (59.6)	NM	40.17 (28.74)
	Eos	188 (82.28)	64.46 (9.19)	NM	86 (37.72)	NM	34.7 (61.7)
	Non-eos	118 (81.48)	64.78 (9.27)	NM	111 (76.55)	NM	35.6 (26.12)
Prins et al. (7)	Eos	23 (58.87)	70.4 (8.7)	25.3 (5.0)	9 (23.08)	NM	40 (7.75)
	Non-eos	78 (46.43)	69.7 (11.5)	24.9 (5.3)	60 (35.71)	NM	40 (6.25)
Roche et al. (20)	Eos	1,594 (77.83)	64.8 (7.73)	NM	771 (37.65)	1,277 (62.35)	NM
	Non-eos	951 (73.10)	64.2 (7.86)	NM	556 (42.74)	745 (57.26)	NM
Saltürk et al. (21)	Eos	51 (82.25)	67 (6)	23 (1.75)	26 (41.94)	15 (24.19)	40 (7.5)
	Non-eos	471 (80.51)	69 (4.25)	23 (2.25)	206 (35.21)	177 (30.26)	43 (7.5)
Serafino Agrusa tet al. (26)	Eos	18 (90)	72.9 (8.6)	31.9 (7.8)	6 (30)	14 (70)	NM
	Non-eos	73 (65.19)	73.3 (9.2)	25.7 (5.9)	46 (41.1)	66 (58.9)	NM
Vedel Krogh et al. (27)	Eos	2,486 (54)	64 (14.07)	25.2 (3.7)	1,661 (36)	NM	30 (22.22)
	Non-eos	1,124 (43)	64 (13.33)	24.7 (3.63)	1,042 (40)	NM	30 (21.48)
Watz et al. (22)	Eos	1,074 (82.17)	64.1 (8.6)	NM	411 (31.45)	896 (68.55)	NM
	Non-eos	915 (82.21)	63.5 (8.4)	NM	402 (36.12)	711 (63.88)	NM
Zysman et al. (23)	Eos	162 (72.6)	62 (3.75)	25.3 (1.88)	64 (28.70)	144 (64.57)	36.0 (7.5)
	Non-eos	168 (71.49)	62 (3.75)	24.2 (1.8)	79 (33.62)	146 (62.13)	37.1 (7.5)

*Eos, eosinophilic COPD; Non-eos, non-eosinophilic COPD; SD, standard deviation; NM, not mentioned; n, numbers*.

**Table 3:** Baseline characteristics of patients in each enrolled trial.

**References**	**Subtype**	**GOLD stage I (*n*, %)**	**GOLD stage II (*n*, %)**	**GOLD stage III (*n*, %)**	**GOLD stage IV (*n*, %)**	**FEV1% (mean, SD)**	**Ischemic heart disease (*n*, %)**	**Hypertension (*n*, %)**	**Diabetes (*n*, %)**
Bafadhel et al. (10)	Eos	NM	NM	NM	NM	44.9 (1.9)	29 (46.77)	NM	9 (14.52)
	Non-eos	NM	NM	NM	NM	40.7 (1.4)	80 (44.2)	NM	13 (7.18)
Barnes et al. (17)	Eos	NM	NM	NM	NM	44.2 (9.45)	NM	NM	NM
	Non-eos	NM	NM	NM	NM	43.9 (9.44)	NM	NM	NM
Barnes et al. (18)	Eos	3 (0.08)	1,882 (49.71)	1,596 (42.16)	305 (8.06)	NM	NM	NM	NM
	Non-eos	3 (0.12)	1,226 (47.19)	1,131 (43.53)	238 (9.16)	NM	NM	NM	NM
Çoban Agca et al. (13)	Eos	NM	NM	NM	NM	NM	32 (5.06)	45 (7.11)	12 (1.90)
	Non-eos	NM	NM	NM	NM	NM	29 (3.38)	35 (4.07)	20 (2.33)
Couillard et al. (9)	Eos	5 (9.09)	28 (50.91)	15 (27.27)	7 (12.73)	53.3 (19.2)	16 (29.09)	NM	11 (21.82)
	Non-eos	8 (7.14)	52 (46.43)	44 (39.29)	8 (7.14)	51.6 (17.2)	45 (41.07)	NM	29 (25.89)
DiSantostefano et al. (14)	Eos	341 (53.79)	247 (38.96)	45 (7.1)	1 (0.16)	NM	50 (7.89)	278 (43.85)	81 (12.78)
	Non-eos	172 (54.78)	127 (40.45)	15 (4.78)	0 (0)	NM	24 (7.64)	121 (38.54)	48 (15.29)
Duman et al. (24)	Eos	NM	NM	NM	NM	NM	16 (4.6)	44 (12.5)	45 (12.8)
	Non-eos	NM	NM	NM	NM	NM	59 (4.4)	166 (12.3)	124 (9.2)
Hastie et al. (8)	Eos	425 (34.36)	153 (12.37)	359 (29.02)	200 (16.17)	74.2 (9.95)	NM	NM	NM
	Non-eos	505 (40.01)	150 (11.89)	323 (25.59)	190 (15.06)	77.7 (10.13)	NM	NM	NM
Iqbal et al. (19)	Eos	0 (0)	1,133 (46.49)	1,040 (42.68)	256 (10.5)	47.8 (13)	NM	NM	NM
	Non-eos	0 (0)	1,033 (46.74)	955 (43.21)	231 (10.45)	47.5 (12.8)	NM	NM	NM
Kang et al. (12)	Eos	NM	NM	NM	NM	NM	8 (4.52)	61 (34.46)	35 (19.77)
	Non-eos	NM	NM	NM	NM	NM	16 (4.21)	145 (38.16)	75 (19.74)
Pascoe et al. (11)	Eos	NM	NM	NM	NM	45.38 (13.26)	NM	NM	NM
	Non-eos	NM	NM	NM	NM	45.58 (13.72)	NM	NM	NM
Pavord et al. (25)	Eos	NM	NM	NM	NM	39.4 (8.5)	NM	NM	NM
	Non-eos	NM	NM	NM	NM	39.06 (8.7)	NM	NM	NM
	Eos	NM	NM	NM	NM	51.13 (14.23)	NM	NM	NM
	Non-eos	NM	NM	NM	NM	50.34 (14.2)	NM	NM	NM
	Eos	NM	NM	NM	NM	56.88 (13.21)	NM	NM	NM
	Non-eos	NM	NM	NM	NM	55.82 (10.64)	NM	NM	NM
Prins et al. (7)	Eos	NM	NM	NM	NM	50.6 (16.0)	NM	NM	NM
	Non-eos	NM	NM	NM	NM	44.6 (16.6)	NM	NM	NM
Roche et al. (20)	Eos	0 (0)	695 (33.94)	1,182 (57.71)	154 (7.57)	NM	NM	NM	NM
	Non-eos	0 (0)	425 (32.67)	764 (58.72)	101 (7.76)	NM	NM	NM	NM
Saltürk et al. (21)	Eos	NM	NM	NM	NM	NM	NM	20 (32.26)	15 (24.19)
	Non-eos	NM	NM	NM	NM	NM	NM	231 (39.66)	112 (19.15)
Serafino Agrusa et al. (26)	Eos	NM	2 (12)	3 (18)	12 (70)	44.9 (6)	8 (40)	17 (85)	8 (40)
	Non-eos	NM	9 (9)	13 (13)	77 (78)	46.1 (14.2)	26 (23)	73 (77)	24 (21)
Vedel Krogh et al. (27)	Eos	2,122 (44.76)	2,062 (45)	423 (9)	0	78 (18.52)	462 (10)	1,212 (26)	92 (2)
	Non-eos	1,222 (45.48)	1,170 (45)	226 (8)	0	79 (18.52)	185 (7)	671 (26)	67 (3)
Watz et al. (22)	Eos	3 (0.22)	5 (0.38)	786 (60.14)	512 (39.17)	34.8 (11.3)	NM	NM	NM
	Non-eos	0 (0)	4 (0.36)	695 (62.44)	411 (36.93)	33.8 (10.6)	NM	NM	NM
Zysman et al. (23)	Eos	NM	NM	NM	NM	52 (7.75)	25 (11.21)	NM	18 (8.07)
	Non-eos	NM	NM	NM	NM	51 (9)	27 (11.49)	NM	39 (16.60)

*Eos, eosinophilic COPD; Non-eos, non-eosinophilic COPD; SD, standard deviation; NM, not mentioned; n, numbers*.

**Table 4:** The results of the risk of bias assessment.

**References**	**Selection of the study groups**	**Comparability of the groups**	**Ascertainment of the exposure or outcome**	**Total score**	**Risk of bias**
Bafadhel et al. (10)	4	1	3	8	Low
Barnes et al. (17)	4	2	3	9	Low
Barnes et al. (18)	4	2	3	9	Low
Çoban Agca et al. (13)	4	2	3	9	Low
Couillard et al. (9)	4	1	3	8	Low
DiSantostefano et al. (14)	4	2	3	9	Low
Duman et al. (24)	4	2	3	9	Low
Hastie et al. (8)	4	2	3	9	Low
Iqbal et al. (19)	4	2	3	9	Low
Kang et al. (12)	4	2	3	9	Low
Pascoe et al. (11)	4	1	3	8	Low
Pavord et al. (25)	4	2	3	9	Low
	4	2	3	9	Low
	4	2	3	9	Low
Prins et al. (7)	4	1	3	8	Low
Roche et al. (20)	4	1	3	8	Low
Saltürk et al. (21)	4	1	3	8	Low
Serafino Agrusa et al. (26)	4	2	3	9	Low
Vedel Krogh et al. (27)	4	2	3	9	Low
Watz et al. (22)	4	1	3	8	Low
Zysman et al. (23)	4	2	3	9	Low

### Heterogeneity

No heterogeneity was observed regarding sex, ex-smoker status, ischemic heart disease, or GOLD stage. In contrast, significant statistical heterogeneities were found in the analysis of age, BMI, current-smoker, pack-years smoked, ppFEV_1_, hypertension, and diabetes (*I*^2^ = 76%, MD −0.33, −0.73–0.07, *P* = 0.10; *I*^2^ = 91%, MD 0.70, 0.27–1.12, *P* = 0.001; *I*^2^ = 96%, OR 0.78, 0.59–1.02, *P* = 0.07; *I*^2^ = 92%, MD 0.52, −1.62–2.67, *P* = 0.63; *I*^2^ = 96%, MD 0.34, −1.03–1.71, *P* = 0.62; *I*^2^ = 51%, OR 1.10, 0.91–1.33, *P* = 0.32; *I*^2^ = 60%, OR 0.99, 0.75–1.30, *P* = 0.93) (Figures 1, 3, 4; Appendix Figures S1, S3, S4, S6, S7). Sensitivity analysis was performed to assess whether any study biased the overall results. The overall effect and summary MDs or ORs were recalculated after removing each study one at a time. This analysis revealed the constancy of the results of age, BMI, current-smoker, pack-years smoked, ppFEV_1_, and hypertension, as the sum MDs or ORs were uniform and without obvious variation, and the total effects (*P*-values) did not reveal a statistically significant difference (range of recalculated summary MDs or ORs: −0.14 to −0.41; 0.57–0.81; 0.69–0.86; −0.48–0.71; 1.04–1.15; 0.92–1.06). The heterogeneity was clearly reduced for hypertension when the study of Çoban Agca et al. (13) was removed. A non-significant difference was found in the analysis of hypertension after recalculation (*I*^2^ = 28, OR 1.04, 0.89–1.21, *P* = 0.63) (Appendix Figure S14).

### Outcomes

#### Primary Outcome

The prevalence of eosinophilic COPD ranged from 18.84 to 66.88% and the mean prevalence across all studies was 54.95%.

#### Secondary Outcome

##### Demographic Characteristics

**Figure 2:**
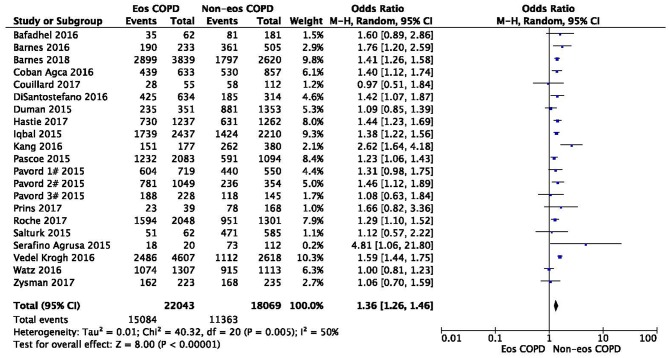
Comparison of gender character between eosinophilic and non-eosinophilic COPD. M.-H., Mantel-Haenszel; CI, confidence interval; Eos, eosinophilic; Non-eos, non-eosinophilic; COPD, chronic obstructive pulmonary disease.

**Figure 3:**
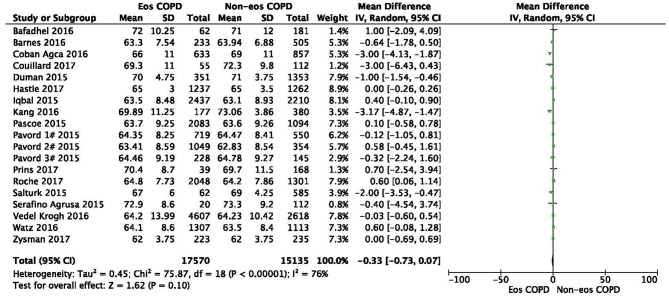
Comparison of age character between eosinophilic and non-eosinophilic COPD. SD, standard derivation; IV, Inverse Variance; CI, confidence interval; Eos, eosinophilic; Non-eos, non-eosinophilic; COPD, chronic obstructive pulmonary disease.

**Figure 4:**
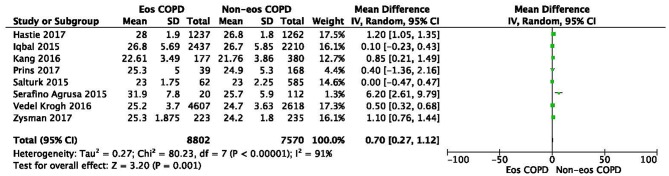
Comparison of BMI character between eosinophilic and non-eosinophilic COPD. SD, standard derivation; IV, Inverse Variance; CI, confidence interval; BMI, body-mass index; Eos, eosinophilic; Non-eos, non-eosinophilic; COPD, chronic obstructive pulmonary disease.

There was a significantly higher rate of male patients and higher BMI in the eosinophilic COPD group (OR 1.36, 95% CI 1.26–1.46, *P* < 0.00001; MD 0.70, 0.27–1.12, *P* = 0.001) (Figures 2, 3). There was no statistically significant difference in age between the two groups (MD −0.33, −0.73–0.07, *P* = 0.10) (Figure 4).

##### Smoking Status

We found a significantly higher rate of ex-smokers in the eosinophilic group (OR 1.23, 1.12–1.34, *P* < 0.0001) (Appendix Figure S2), but no difference was found in the proportion of current smokers or pack-years smoked (OR 0.78, 0.59–1.02, *P* = 0.07; MD 0.52, −1.62–2.67, *P* = 0.63) (Appendix Figures S1, S3).

##### Lung Function

With regard to lung function, no significant difference was found in the ppFEV_1_ between the two groups (MD 0.34, −1.03–1.71, *P* = 0.62) (Appendix Figure S4).

##### Comorbidity

A significantly higher rate of ischemic heart disease was found in the eosinophilic COPD group (OR 1.31, 1.14–1.50, *P* = 0.001) (Appendix Figure S5). However, there was no significant difference in hypertension or diabetes between the groups (OR 1.10, 0.91–1.33, *P* = 0.32; OR 0.99, 0.75–1.30, *P* = 0.93) (Appendix Figures S6, S7).

##### GOLD Stage

A significantly lower rate of GOLD stage I was found in the eosinophilic COPD group (OR 0.84, 0.73–0.96, *P* = 0.01) (Appendix Figure S8). No significant difference was found in the proportion of subjects with GOLD stage II, III, or IV between the two groups (OR 1.04, 0.98–1.09, *P* = 0.17; OR 0.99, 0.94–1.04, *P* = 0.67; OR 1.01, 0.92–1.10, *P* = 0.89) (Appendix Figures S9–S11).

##### Subgroup Analysis

When restricted to different disease statuses, a significantly higher proportion of male patients was observed in both the stable and acute exacerbation phases of COPD in the eosinophilic group (*n* = 34,507, OR 1.36, 95% CI 1.26–1.47, *P* < 0.00001; *n* = 5605, OR 1.39, 95% CI 1.11–1.73, *P* = 0.004) (Appendix Figure S12). The subgroup analysis found that subjects in the eosinophilic group were significantly younger when restricting the analysis to the acute exacerbation phase. No difference was found between groups in the stable phase (*n* = 5605, MD −1.38, −2.34 to −0.42, *P* < 0.0001; n = 27,100, MD 0.16, −0.02–0.33, *P* = 0.08) (Appendix Figure S13).

## Discussion

This comprehensive systematic review and meta-analysis investigated the prevalence and baseline clinical characteristics of eosinophilic COPD. The prevalence of eosinophilic COPD ranged from 18.84 to 66.88%, with an average prevalence of 54.95% across all studies. The prevalence of COPD varied greatly owing to differences in diagnostic criteria, as well as survey and analytical methods. Reasons for the large range in the prevalence of eosinophilic COPD may be similar, except for the effect of different races, regions, and countries (28). In Japan, there was a tendency to exclude patients with any feature of asthma from the diagnosis of COPD, especially in younger patients with milder forms of the disease. This then leads to a low diagnostic rate of eosinophilic COPD (29–32).

In this study, we found that male patients are more at risk for eosinophilic COPD (OR 1.36, 95% CI 1.26–1.46, *P* < 0.00001) (Figure 2). Sex is one of the most fundamental and defining features of subpopulations in human beings. A higher absolute eosinophil count and eosinophil percentage were observed in men in an observational study; however, the number of participants enrolled was relatively low (476) (33). This may suggest that men are prone to having higher eosinophil levels, and that eosinophilic inflammation increases the risk of progression to COPD in men. More trials are needed, however, to verify this hypothesis. A higher BMI was also observed in the eosinophilic group (MD 0.70, 0.27–1.12, *P* = 0.001) (Figure 3). Our result was consistent with the result of a longitudinal analysis, which revealed that COPD patients with persistent eosinophil levels of >2% had fat-free mass (2). No significant difference in age was found between the two groups (MD −0.33, −0.73–0.07, *P* = 0.10) (Figure 4). Considering that the primary analysis in our study was aimed at identifying the characteristics of the subtypes of COPD, there is no prior relevant information that can be referenced. The mechanisms for these differences remain unclear.

Regarding smoking status, we found a significant difference in the proportion of ex-smokers between the two groups (OR 1.23, 1.12–1.34, *P* < 0.0001) (Appendix Figure S2), but no difference in the proportion of current smokers or in pack-years smoked (OR 0.78, 0.59–1.02, *P* = 0.07; MD 0.52, −1.62–2.67, *P* = 0.63) (Appendix Figures S1, S3). Pooled analysis showed that the prevalence of ex-smokers was higher in patients with eosinophilic COPD. The inflammation detected in the respiratory tract may be a modified inflammatory response to chronic irritants, such as cigarette smoke. The presence of persistent lung inflammation after smoking cessation remains unknown; even perturbations and autoantigens in the lung microbiome may play a role (34, 35). We hypothesize that smoking may induce eosinophilic inflammation and that the inflammation persists even after smoking cessation, although more research is needed to confirm this hypothesis.

In terms of lung function, no significant difference was found in the ppFEV_1_ (MD 0.34, −1.03–1.71, *P* = 0.62) (Appendix Figure S4). A significantly lower prevalence of GOLD stage I was, however, found in the eosinophilic COPD group (OR 0.84, 0.73–0.96, *P* = 0.01) (Appendix Figure S8). We believe that the mild severity of airflow limitations is more common in non-eosinophilic COPD and rarer in eosinophilic COPD. Our analysis was not consistent with previous findings. In the ECLIPSE cohort study, patients with COPD with persistent eosinophil levels of >2% had a significantly higher ppFEV_1_ (2). In the SPIROMICS study, patients with a lower baseline eosinophil level (<1%) were prone to severe COPD (36). There is no definitive explanation, however, for this problem. Additional studies are needed to further investigate the relationship between eosinophil and FEV_1_ in COPD patients.

There was a significantly lower prevalence of chronic heart failure in the eosinophilic COPD group (OR 0.81, 0.68–0.97, *P* = 0.02) (Appendix Figure S5). No difference in the prevalence of hypertension or diabetes between groups was found. COPD patients often have important concomitant illnesses. The SPIROMICS study suggested a higher incidence of comorbidities (prior heart attack, anemia, diabetes, and chronic heart failure) among COPD patients with eosinophil levels of ≤2% (36). The comorbidities in COPD may be caused by original genetic variances in response to the inhalation of poisonous particles, particularly during smoking (37). More rigorous trials are needed to clarify this issue.

Significant variability in blood eosinophil levels has been shown throughout the course of COPD (38, 39). To investigate the stability of blood eosinophilic inflammation (≥2%), subjects were classified into predominantly (PE), intermittently (IE), and rarely (RE) eosinophilic groups in one study (40). The PE group was characterized by an increased risk of eosinophilic inflammation during exacerbation. The PE group at stable visits and eosinophilia during exacerbation were associated with a minor risk of bacterial infection during exacerbation. Bacterial infection during exacerbation was higher in winter in the PE group. Blood eosinophil counts in the stable status could predict the nature of inflammation during future exacerbations. When combined with an understanding of seasonal variation, this may also provide a basis for the development of new therapy. More research, however, is warranted.

Although blood eosinophil is considered to be a promising biomarker, eosinophil-guided treatment of acute exacerbation of COPD remains an issue. Bafadhel et al. (41) showed that systemic corticosteroid use in a low eosinophil (<2%) group was associated with less improvement in chronic respiratory questionnaire scores and higher treatment failure when compared to the placebo group. On the contrary, Sivapalan et al. (42) reported that, when compared to standard therapy in patients hospitalized for COPD, eosinophil-guided therapy did not lead to a difference in the number of days alive, number of patients discharged from the hospital within 14 days of recruitment, or the risk of treatment failure at 30 days. Future studies on eosinophil-guided therapies are needed.

This study has several strengths. First, it is a comprehensive systematic review and meta-analysis to analyze the prevalence and baseline clinical characteristics of eosinophilic COPD. Additionally, the studies that were included were of high quality. All data were collected at the very beginning of each study, protecting against subsequent interference. Our results are thus highly credible. This study has some limitations. First, the studies that were included were not RCTs. Nonetheless, the extracted data were obtained from RCTs that enrolled a large number of patients with COPD and classified according to eosinophilic and non-eosinophilic inflammation status. Second, given that this analysis is the first to verify the baseline clinical characteristics of eosinophilic COPD, the underlying mechanisms remain unclear. Finally, a proportion of patients had already been treated with corticosteroids and antibiotics in the community. It remains unclear whether and to what extent these therapies affect the eosinophil count. Further research is therefore warranted.

## Conclusions

In conclusion, eosinophilic inflammation is prevalent in COPD. Eosinophilic COPD was more common in men, ex-smokers, subjects with higher BMI, and in those with a high risk of some comorbidity. The group also included a low proportion of patients with mild airflow limitations. Future rigorous prospective trials are needed, particularly in basic research, to further identify the relationship between eosinophil levels and COPD. Additional studies should explore the exact mechanisms that are responsible for the characteristics of eosinophilic COPD.

## Data Availability Statement

The datasets used and/or analyzed during the current study are available from the corresponding author on reasonable request.

## Author Contributions

H-XW and D-YC initiated and coordinated the study. H-XW and K-QZ were responsible for the data collection and data analysis. Studies were reviewed by D-YC. H-XW wrote the first draft of the manuscript. All the authors were involved in the interpretation of the analyses and gave input to the final manuscript.

### Conflict of Interest

The authors declare that the research was conducted in the absence of any commercial or financial relationships that could be construed as a potential conflict of interest.

## Abbreviations

COPD: chronic obstructive pulmonary disease
BMI: body-mass index
ppFEV_1_: percent of predicted forced expiratory volume in 1 s
GOLD: global initiative for chronic obstructive lung disease.
