# Supplementary material for: Prevalence and Baseline Clinical Characteristics of Eosinophilic Chronic Obstructive Pulmonary Disease: A Meta-Analysis and Systematic Review
Source: Front Med (Lausanne). 2019 Dec 10;6:282. doi: 10.3389/fmed.2019.00282 (PMC6916535; doi:10.3389/fmed.2019.00282)
Supplement: Supplementary file 1 [file Data_Sheet_1.docx]

**Appendix Figure S1:** Comparison of current smoker between eosinophilic and non-eosinophilic COPD. M.-H., Mantel-Haenszel; CI, confidence interval; Eos, eosinophilic; Non-eos, non-eosinophilic; COPD, chronic obstructive pulmonary disease.

**Appendix Figure S2:** Comparison of ex-smoker between eosinophilic and non-eosinophilic COPD. M.-H., Mantel-Haenszel; CI, confidence interval; Eos, eosinophilic; Non-eos, non-eosinophilic; COPD, chronic obstructive pulmonary disease.

**Appendix Figure S3:** Comparison of pack-years smoked between eosinophilic and non-eosinophilic COPD. SD, standard derivation; IV, Inverse Variance; CI, confidence interval; Eos, eosinophilic; Non-eos, non-eosinophilic; COPD, chronic obstructive pulmonary disease.

**Appendix Figure S4:** Comparison of FEV_1_% between eosinophilic and non-eosinophilic COPD. SD, standard derivation; IV, Inverse Variance; CI, confidence interval; Eos, eosinophilic; Non-eos, non-eosinophilic; COPD, chronic obstructive pulmonary disease; FEV_1_%, percent of predicted forced expiratory volume in 1s.

**Appendix Figure S5**: Comparison of ischemic heart disease between eosinophilic and non-eosinophilic COPD. M.-H., Mantel-Haenszel; CI, confidence interval; Eos, eosinophilic; Non-eos, non-eosinophilic; COPD, chronic obstructive pulmonary disease.

**Appendix Figure S6:** Comparison of hypertension between eosinophilic and non-eosinophilic COPD. M.-H., Mantel-Haenszel; CI, confidence interval; Eos, eosinophilic; Non-eos, non-eosinophilic; COPD, chronic obstructive pulmonary disease.

**Appendix Figure S7:** Comparison of diabetes between eosinophilic and non-eosinophilic COPD. M.-H., Mantel-Haenszel; CI, confidence interval; Eos, eosinophilic; Non-eos, non-eosinophilic; COPD, chronic obstructive pulmonary disease.

**Appendix Figure S8:** Comparison of GOLD stage I between eosinophilic and non-eosinophilic COPD. M.-H., Mantel-Haenszel; CI, confidence interval; Eos, eosinophilic; Non-eos, non-eosinophilic; COPD, chronic obstructive pulmonary disease; GOLD, Global Initiative for Chronic Obstructive Lung Disease.

**Appendix Figure S9:** Comparison of GOLD stage II between eosinophilic and non-eosinophilic COPD. M.-H., Mantel-Haenszel; CI, confidence interval; Eos, eosinophilic; Non-eos, non-eosinophilic; COPD, chronic obstructive pulmonary disease; GOLD, Global Initiative for Chronic Obstructive Lung Disease.

**Appendix Figure S10:** Comparison of GOLD stage III between eosinophilic and non-eosinophilic COPD. M.-H., Mantel-Haenszel; CI, confidence interval; Eos, eosinophilic; Non-eos, non-eosinophilic; COPD, chronic obstructive pulmonary disease; GOLD, Global Initiative for Chronic Obstructive Lung Disease.

**Appendix Figure S11:** Comparison of GOLD stage IV between eosinophilic and non-eosinophilic COPD. M.-H., Mantel-Haenszel; CI, confidence interval; Eos, eosinophilic; Non-eos, non-eosinophilic; COPD, chronic obstructive pulmonary disease; GOLD, Global Initiative for Chronic Obstructive Lung Disease.

**Appendix Figure S12:** Comparison of disease status subgroup of gender between eosinophilic and non-eosinophilic COPD. M.-H., Mantel-Haenszel; CI, confidence interval; Eos, eosinophilic; Non-eos, non-eosinophilic; COPD, chronic obstructive pulmonary disease.

**Appendix Figure S13:** Comparison of disease status subgroup of age between eosinophilic and non-eosinophilic COPD. SD, standard derivation; IV, Inverse Variance; CI, confidence interval; Eos, eosinophilic; Non-eos, non-eosinophilic; COPD, chronic obstructive pulmonary disease.

**Appendix Figure S14:** Recalculated comparison of hypertension between eosinophilic and non-eosinophilic COPD. M.-H., Mantel-Haenszel; CI, confidence interval; Eos, eosinophilic; Non-eos, non-eosinophilic; COPD, chronic obstructive pulmonary disease.
